# The Source of the Symbolic Numerical Distance and Size Effects

**DOI:** 10.3389/fpsyg.2016.01795

**Published:** 2016-11-21

**Authors:** Attila Krajcsi, Gábor Lengyel, Petia Kojouharova

**Affiliations:** ^1^Department of Cognitive Psychology, Institute of Psychology, Eötvös Loránd UniversityBudapest, Hungary; ^2^Department of Cognitive Science, Central European UniversityBudapest, Hungary; ^3^Doctoral School of Psychology, Eötvös Loránd UniversityBudapest, Hungary

**Keywords:** numerical cognition, numerical distance effect, numerical size effect, analog number system, discrete semantic system

## Abstract

Human number understanding is thought to rely on the analog number system (ANS), working according to Weber’s law. We propose an alternative account, suggesting that symbolic mathematical knowledge is based on a discrete semantic system (DSS), a representation that stores values in a semantic network, similar to the mental lexicon or to a conceptual network. Here, focusing on the phenomena of numerical distance and size effects in comparison tasks, first we discuss how a DSS model could explain these numerical effects. Second, we demonstrate that the DSS model can give quantitatively as appropriate a description of the effects as the ANS model. Finally, we show that symbolic numerical size effect is mainly influenced by the frequency of the symbols, and not by the ratios of their values. This last result suggests that numerical distance and size effects cannot be caused by the ANS, while the DSS model might be the alternative approach that can explain the frequency-based size effect.

## An Alternative To The Analog Number System

**FIGURE 1:**
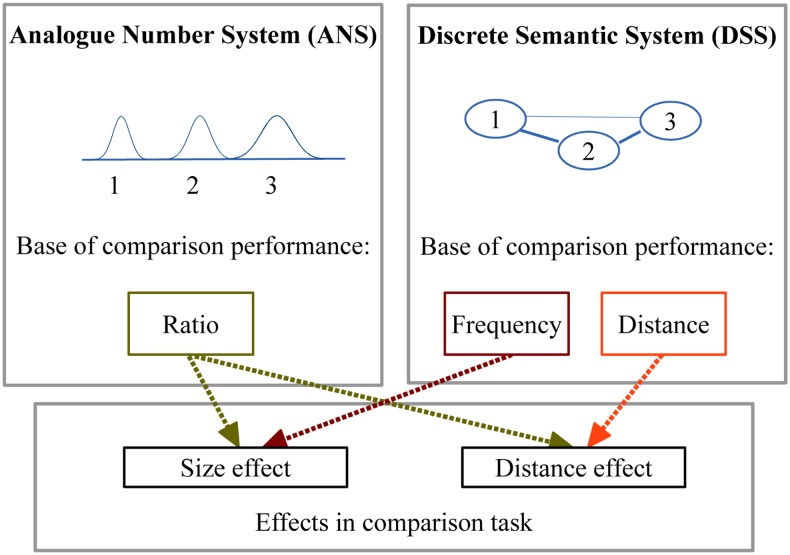
**The sources of the distance and size effects according to the two models**.

**FIGURE 2:**
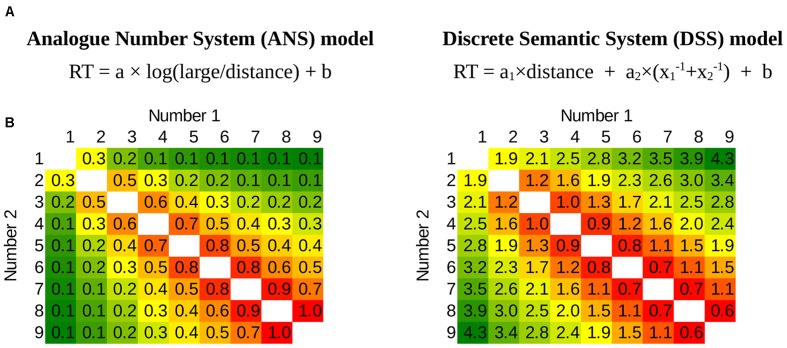
**(A)** Reaction time (RT) function for the ANS model (based on Crossman, 1955; Moyer and Landauer, 1967) (left) and a hypothetical RT function for the DSS model where the reaction time is proportional to a combination of the specific forms of the distance and the frequencies of the numbers (right). Notations: large: larger number; distance: distance between the two numbers; x_1_ and x_2_: the two numbers; a, a_1_, a_2_ and b are free parameters. **(B)** The prediction of the models on a full stimulus space in a number comparison task of numbers between 1 and 9. Numbers 1 and 2 are the two values to be compared. Green denotes fast responses, red denotes slow responses (note that numerically the ANS function increases, and the DSS function decreases toward the high ratio, but the direction is irrelevant in the linear fit below). The distance effect can be seen as the gradual change when getting farther from the top–left bottom–right diagonal, and the size effect is seen as the gradual change from top–left to bottom–right. In the figures the parameters *a* and *a_2_* are set to 1, *a_1_* is 0.4, and parameter *b* is set to 0.

According to the current models understanding numbers is supported by an evolutionary ancient representation shared by many species (Dehaene et al., 1998; Gallistel and Gelman, 2000; Hauser and Spelke, 2004), the analog number system (ANS). One defining feature of the ANS is that it works similarly to some perceptual representations in which the ratio of the stimuli’s intensity determines the performance (Weber’s law) (Moyer and Landauer, 1967; Walsh, 2003; Cantlon et al., 2009). Two critical phenomena supporting the ratio based performance are the distance and the size effects: when two numbers are compared, the comparison is slower and more error prone when the distance between the two values is smaller (distance effect) or when the two numbers are larger (size effect), (Moyer and Landauer, 1967) (**Figures 1** and **2**). Thus, in the literature, the numerical distance and size effects are considered to be the sign of an analog noisy numerical processing system working according to Weber’s law. The distance and the size effects are observable both in non-symbolic and symbolic number processing, reflecting that the same type of system processes numerical information, independent of the number notations (Dehaene, 1992; Eger et al., 2003).

However, the distance and size effects in symbolic comparison can also be explained by a different representation. Quite intuitively, one might think that symbolic and abstract mathematical concepts, like numbers could be handled by a discrete semantic system (DSS), similar to conceptual networks or to the mental lexicon, i.e., representations that process symbolic and abstract concepts. In this DSS model, numbers are stored in a network of nodes, and the strength of their connections is proportional to the strength of their semantic relations. We propose that this DSS account could be responsible for symbolic number processing; whereas non-symbolic number processing is still supported by the ANS (see some additional details about the relation of the two models below). The main aim of the present study is to investigate the feasibility of the DSS model as a comprehensive explanation of the symbolic numerical effects, and to contrast it with the ANS model.

### DSS Explanation for the Distance and Size Effects

How can a DSS explain the symbolic numerical distance and size effects? (1) Regarding the distance effect, the strength of the connections between the nodes can produce an effect which is proportional to their strength, and since in a network storing numbers the strength of the connections is proportional to the numerical values and numerical distance, this system could produce a numerical distance effect. In fact, a similar semantic distance effect was shown in a picture naming task (Vigliocco et al., 2002): Naming time slowed down when the picture of the previous trial was semantically related to the present picture, and a small semantic distance between the previous and the actual word caused stronger effect than a large semantic distance, similar to the numerical distance effect^1^. This semantic distance effect cannot be the result of a continuous representation similar to the ANS, because the stimuli were categorical (e.g., finger, car, smile, etc.)^2^. Thus, a discrete representation potentially can produce a numerical distance effect. Several mechanisms can be imagined how a numerical distance effect is generated. One can imagine that the semantic distance information, that can be revealed in a semantic priming, could generate a distance effect. Alternatively, it is possible that the strength of the association between the numbers and the large–small categories create the numerical distance effect (Verguts and Fias, 2004; Verguts et al., 2005). Here, we do not want to specify the exact mechanism behind the numerical distance effect, but only propose that several possible mechanisms are already available in the literature. (2) Turning to the size effect, this effect also could be generated by a DSS. It is known that smaller numbers are more frequent than larger numbers, and the frequency of a number is proportional to the power of its value (Dehaene and Mehler, 1992). Since the numbers observed more frequently could be processed faster, the size effect could result from this frequency pattern^3^. Thus, the DSS model can also explain the appearance of distance and the size effects (**Figure 1**).

### DSS Explanation for Other Numerical Effects

Whereas in the present work we focus on the DSS explanation of the distance and size effects, the DSS explanation can be readily extended to other effects, too, and it can be a comprehensive model of symbolic number processing. The following details can demonstrate that despite its radical difference from the ANS model, DSS might be a viable option to explain symbolic numerical phenomena. Many of these explanations have already been proposed in the literature, although these explanations usually focused on single specific phenomena, and they did not offer a comprehensive model.

Several interference effects can be explained in the DSS framework. For example, the SNARC effect (interference between numerical value and response location in a task) was originally interpreted as the interference of the ANS’s spatial property and the response locations (Dehaene et al., 1993), however, it is also possible that the effect is the result of the interference of the left-right and large-small nodes in a semantic network similar to the DSS (Proctor and Cho, 2006; Leth-Steensen et al., 2011; Patro et al., 2014; Krajcsi et al., unpublished). Similarly, while the size congruency effect (Stroop-like interference between the numerical value and the physical size of symbols; Henik and Tzelgov, 1982) can be thought of as an interference between the ANS and a representationally similar analog size representation, it can also be thought of as an interference between the many-few and the physically large-physically small nodes.

While there are many empirical and theoretical works in the literature that support the ANS model, in fact there are only a handful effects that are cited to support the ANS model, and we propose that most of these effects (in fact to our knowledge all of them at the moment) can also be explained by the DSS. While mostly it would not be too difficult to find DSS explanations for different phenomena, in the present work we only focus on the numerical distance and size effects in comparison tasks.

### Different Representations for Symbolic and Non-symbolic Numbers

As it was mentioned above, the DSS model can only account for symbolic number processing. Clearly, there are cases when the DSS cannot handle numerical information, for example, when the symbolic mental tools are not available, like in the case of infants (Feigenson et al., 2004), animals (Hauser and Spelke, 2004), or adults living in a culture without number words (Gordon, 2004; Pica et al., 2004), therefore, the ANS seems to be a sensible model to explain these non-symbolic phenomena. It also seems reasonable that because of their representational structure, the two systems could be specialized for different forms of numbers: The DSS could be responsible for the precise and symbolic numbers, while the ANS could process the approximate non-symbolic stimuli.

This idea of different representations for symbolic and non-symbolic numbers is supported by the increasing number of findings in the literature, suggesting that symbolic and non-symbolic number processing is supported by different representations. For example, it has been shown that performance of the symbolic and non-symbolic number comparison tasks do not correlate in children (Holloway and Ansari, 2009; Sasanguie et al., 2014), and in an fMRI study the size of the symbolic and non-symbolic number activations did not correlate (Lyons et al., 2015). As another example, whereas former studies found common brain areas activated by both symbolic and non-symbolic stimuli (Eger et al., 2003; Piazza et al., 2004), later works with more sensitive methods found only notation-dependent activations (Damarla and Just, 2013; Bulthé et al., 2014, 2015). According to an extensive meta-analysis, although it was repeatedly found that simple number comparison task (the supposed sensitivity of the ANS) correlates with mathematical achievement, it seems that non-symbolic comparison correlates much less with math achievement, than symbolic comparison (Schneider et al., 2016). In another example, Noël and Rousselle (2011) found that whereas older than 9- or 10-year-old children with developmental dyscalculia (DD) perform worse in both symbolic and non-symbolic tasks than the typically developing children; younger children with DD perform worse than control children only in the symbolic tasks, but not in the non-symbolic tasks. The authors concluded that the deficit in DD can be explained in the terms of two different representations: The deficit is more strongly related to the symbolic number processing, and the impaired non-symbolic performance is only the consequence of the symbolic processing problems. See a more extensive review of similar findings in Leibovich and Ansari (2016). All of these findings are in line with the present proposal, suggesting that symbolic and non-symbolic numbers are processed by different systems.

### Related Models for Symbolic Number Processing

There are former models in the literature that are potential alternatives to the ANS model, and some of those models can be fitted into a DSS framework, or they could be considered as implementations of more specific aspects of the DSS account.

Verguts et al. (2005) and Verguts and Van Opstal (2014) proposed a connectionist model describing several phenomena of number processing and more generally several phenomena of ordinal information processing. According to their simulations and experiments, this model offers a superior description of number naming, parity judgment and number comparison than the ANS model, and their model can also explain non-numerical order processing phenomena. Their model includes a hidden layer representing the values of the numbers in a place-code with a fixed width of noise. This means that the nodes of the hidden layer represent numbers on a linear scale, and a number most strongly activates the node mainly representing that number, but additional activation also can be found in the neighboring nodes. The distance these additional activations can reach to do not depend on the source number, i.e., the noise has a fixed width. Although the authors suggest that this model implements an analog representation, it contradicts the ANS model, because on a linear inner scale the size of the noise is not proportional to the size of the number, and relatedly it could not generate ratio-based performance. In line with this representational issue, the model in itself cannot produce a size effect, and an uneven frequency of numbers should be introduced to generate the numerical size effect (Verguts and Fias, 2004; Verguts et al., 2005), questioning whether this model can be seen as an ANS-like model. However, we propose here that the model can be interpreted as a discrete symbolic representation: Activation in the neighboring nodes is not the noise of that representation but it is a spreading activation in the hidden layer. With this alternative interpretation the model can be seen as a specific implementation of the discrete symbolic system when stimuli are arranged as an ordered list. Note that in their model the comparison distance effect is not explained by the spreading activation, but by the connection weights between the value nodes and the response nodes (Verguts et al., 2005; Verguts and Van Opstal, 2014). This model as a potential DSS implementation can give a more precise description for a whole range of phenomena, the ANS model could not account for, thus, strengthening the DSS explanation of symbolic number processing.

Tracking a different line, Henik and Tzelgov (1982) investigated automatic processing of numbers with the size congruency effect (interference between the physical size and numerical value properties of the stimuli). Based on their results they suggested that some basic elements (primitives) are stored in the long term memory, e.g., integers from 1 to 9 and the number 0 (Pinhas and Tzelgov, 2012), while other numbers are not stored as basic elements, e.g., negative numbers and ratios (Kallai and Tzelgov, 2009; Tzelgov et al., 2009). The basic elements or primitives can be considered as the nodes of the DSS: These basic elements could be the values that are stored in the nodes of the network, while other numbers are the combination of the primitives, somewhat similar to the relation of words and sentences. Also, the size congruency effect can be used as a method to find whether a number is stored as a unit in the DSS.

### Possible Quantitative Descriptions of Symbolic Comparison Performance in the DSS Model

While the DSS model can explain why the numerical distance and size effects appear in a comparison task, the ANS model not only suggests that there should be numerical distance and size effects, but it offers a quantitative description for the performance. For example, Moyer and Landauer (1967) proposed that the reaction time of a comparison task is proportional to the following function: *K × log (large_number/(large_number – small_number))*. (See Dehaene, 2007 for a more detailed description of the ANS predictions for behavioral numerical decisions.)

One of the next challenges for the DSS model is to find a quantitative description similar to the ANS model. As in the ANS model where the details of the model were borrowed from psychophysics models, we borrow the details of the DSS model from psycholinguistics and semantic network models. Unfortunately, whereas in many cases the psychophysics models offer quantitative descriptions of the performance (Dehaene, 2007; Kingdom and Prins, 2010), the bases of the DSS model do not have consensual quantitative descriptions. Additionally, our description does not build upon a detailed working model with specific mechanisms (e.g., as it was mentioned, there could be different candidates that could generate the distance effect), but a functional description of these potential effects are given here. Thus, our quantitative proposal is unavoidably speculative, although there are some constrains we can build upon. First, one term of this quantitative description should depend on the distance between the two values. Second, another term should depend on the frequencies of the values, where the frequency of the number is the power of that number (Dehaene and Mehler, 1992). Current theoretical considerations do not specify what distance and size functions should be used, how the frequency of the two numbers should be combined, and how exactly the two terms create performance, thus these details are unavoidably speculative at the moment, and future work can refine the versions offered here. However, based on these few starting points, a number of alternative versions of the DSS model can be created, and many of them display a qualitatively similar pattern of number comparison performance. One simple example is displayed on **Figure 2**, where, as the mathematically simplest version, the distance effect is a linear function, the frequencies of the numbers are summed up, and the distance and size components are added up. This DSS-motivated function creates a qualitatively very similar pattern to the function of the ANS model: Looking at the patterns, the two models are rather similar, also reflected in the high correlation between the two models (*r* = -0.89). Thus, one can create a hypothetical quantitative description based on the DSS account that seemingly can explain the comparison performance in a similar way as the ANS model^4^.

In the first section, so far we have introduced the DSS model, an alternative to the ANS explanation of number processing, where the basic building blocks of the representation are nodes with appropriate connections. We have reasoned that the DSS framework can be a comprehensive explanation of symbolic number processing. While focusing on the comparison distance and size effects, we have demonstrated that the DSS model is capable of giving as appropriate a description of the comparison performance as the ANS model. In the following parts we turn to empirical tests. First, we investigate which model describes better an Indo-Arabic comparison task. Then, we investigate a very specific aspect of number comparison where the two models have clearly different predictions: Whether the size effect depends on the frequency of the numbers (predicted by the DSS model) or on the ratio of the numbers (predicted by the ANS model).

## Experiment 1 – Goodness Of The Two Quantitative Description Of The Models In Indo-Arabic Comparison

After creating a quantitative description for the DSS model, we can contrast the two models, testing which model (**Figure 2**) fits better the empirical data in an Indo-Arabic number comparison task. Although the two models strongly correlate, and the differences between them are subtle, still, there are differences between them, and it is possible that those differences are detectable in a simple comparison task, supposing that the noise is relatively low.

### Methods

#### Participants

Twenty university students participated in the study. Pilot studies with Indo-Arabic and new symbols (see also the second experiment) aiming to refine the applied paradigms revealed that the main effects to be observed can be detected reliably with a sample size of around 20. After excluding two participants because of a higher than 5% error rate, the sample included 18 participants (15 females, mean age 21.5 years, standard deviation 2.8 years). All studies reported here were carried out in accordance with the recommendations of the Department of Cognitive Psychology ethics committee with written informed consent from all subjects. All subjects gave written informed consent in accordance with the Declaration of Helsinki.

#### Stimuli and Procedure

The participants compared Indo-Arabic number pairs. In a trial two numbers between 1 and 9 were shown until response and the participants chose the larger one. All possible number pairs including numbers between 1 and 9 were shown 10 times, excluding ties, resulting in 720 trials. Presentation of the stimuli and measurement of the responses were managed by the PsychoPy software (Peirce, 2007).

#### Analysis Methods

In the analysis, we contrasted the two models with analyzing the reaction times, the error rates, and the diffusion analysis drift rates. (1) Reaction time analysis was used, because response latency may be a more sensitive measurement than the error rate, and the results are comparable with many former results, including the seminal Moyer and Landauer (1967) paper. However, there is no strong consensus which function could describe the ANS model (see the applied version below). (2) Error rate analysis was chosen, because the function describing error rate performance is well established (Dehaene, 2007; Kingdom and Prins, 2010), even if the measurement is not as sensitive as the reaction time data. (3) Finally, drift rate was applied, because diffusion analysis is thought to be more sensitive than the error rate or the reaction time, although its parameter recover methods could be debated. In the recent decades, the diffusion model and related models became increasingly popular to describe simple decision processes (Smith and Ratcliff, 2004; Ratcliff and McKoon, 2008). In the diffusion model, decision is based on a gradual accumulation of evidence offered by perceptual and other systems. Decision is made when an appropriate amount of evidence is accumulated. Reaction time and error rates partly depend on the quality of the information (termed the drift rate) upon which the evidence is built. Importantly for our analysis, observed reaction time and error rate parameters can be used to recover the drift rates (Ratcliff and Tuerlinckx, 2002; Wagenmakers et al., 2007). Drift rates can be more informative than the error rate or reaction time in them, because drift rates reveal the sensitivity of the background mechanisms more directly (Wagenmakers et al., 2007).

Because different versions of the ANS models and the DSS models can be proposed, multiple versions of the models were tested, when it was necessary. For the ANS model the following functions were used in the analysis. (1) Regarding the reaction time analysis, although there are several considerations how to describe the reaction time function of continuous perceptual comparisons (Crossman, 1955; Welford, 1960; Dehaene, 2007), it is not straightforward which version should be applied to describe the ANS model (Kingdom and Prins, 2010). First, we used the version used by Moyer and Landauer (1967), displayed in **Figure 2**. Second, we applied the RT α 1/(log(large/small)) function suggested by Crossman (1955), which function he finds to be more superior compared to the previous function. (2) For the error rate analysis we used the ANS model described in Dehaene (2007, equation 10), which supposes a linear scaling in the ANS,

$$p_{c\,o\,r\,r\,e\,c\,t}\,\left(n\,1,n\,2\right)=\int_{o}^{+\alpha }\frac{e^{-\frac{1}{2}\,{\left(\frac{x-\left(r-1\right)}{w\,\sqrt{1+r^{2}}}\right)}^{2}}}{\sqrt{-2\,\pi \,w}\,\sqrt{1+r^{2}}}\mathrm{d}x$$

where *n_1_* and *n_2_* are the two numbers to be compared, *r* is the ratio of the larger and the smaller number, and *w* is the Weber ratio. (3) Regarding the drift rates, in the ANS model the stored values to be compared can be conceived as two random Gaussian variables, and the difficulty of the comparison might depend on the overlap of the two random variables: Larger overlap leads to worse performance (see the detailed mathematical description in Dehaene, 2007). It is supposed that in a comparison task the drift rate depends purely on the overlap of the two random variables (Palmer et al., 2005; Dehaene, 2007). According to the current theories, *drift_rate = k × task_difficulty*, (Palmer et al., 2005; Dehaene, 2007), or it could also include a power term as a generalization, *drift_rate = k × task_difficulty^β^*, although the exponent is often close to 1, thus the first, proportional model approximates the second, power model. Task difficulty is measured as stimulus strength, which is calculated with the *distance/large_number* function as suggested by Palmer et al. (2005) for psychophysics comparison. Because in an analog representation as the task becomes more difficult (i.e., the two stimuli become indistinguishable) the drift rate tends to zero, in the linear fit this means that the intercept is forced to be zero. To summarize, the *drift_rate* = *k* × *distance*/*large_number* function was used in the drift rate analysis fit for the ANS model.

For the DSS model, two versions were used in the analysis. First, the simple linear version was applied, as described in **Figure 2**. Additionally, a logarithmic version of the DSS model was also used, in which the logarithm of the two terms are used, i.e., RT α log(distance) + log(x_1_^-1^× x_2_^-1^). This logarithmic version seems reasonable, because strictly speaking the distance effect cannot be linear, since that would result in negative reaction time or error performance for sufficiently large distances (even if the linear version could be an appropriate approximation). Additionally, the logarithmic distance effect is partly confirmed by the second experiment and by the inspection of the residuals (results not presented here).

#### Detecting the Distance and Size Effects

The present analysis is not relevant in contrasting the ANS and DSS models, but in the second and third experiments the existence of the numerical distance and size effects was tested, and the same analysis was run in the present experiment, to be able to use these results as a point of reference. The slopes of the specific effects were tested (1) with multiple linear regressions, and (2) with simple linear regressions.

##### Methods for multiple linear regression

**FIGURE 3:**
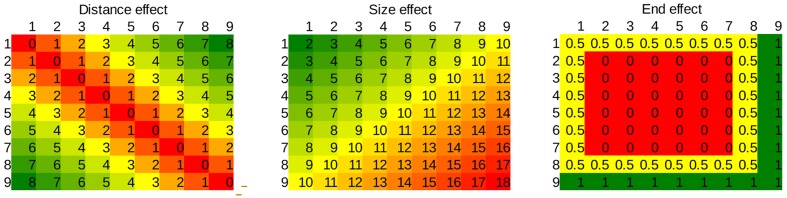
**Values of the three regressors applied in the multiple linear regression in the whole stimulus space**.

Average error rates and median reaction times of the correct responses were calculated for each number pair for each participant. Error rates and reaction times were fitted with two regressors for all participants: (a) distance effect (the absolute difference of the two values), (b) size effect (the sum of the two values). See the values of the regressors for the whole stimulus space on **Figure 3**. (The end effect regressor is used only in the second and third experiments.) This analysis gives a more stable result compared to the more commonly applied simple linear regression analysis (see below). The weights of the regressors were calculated for each participant in both error rates and reaction times, and all regressors’ values were tested against zero.

##### Methods for simple linear regression

To test our data with a more commonly applied simple linear regression, all multiple linear regression analyses were retested. For the distance effect the trials were grouped according to distance (absolute difference between the two numbers) for all participants. For the size effect the trials were grouped according to the sum of the two numbers, excluding trials with distance larger than 3. The latter was necessary, because otherwise the specific shape of the stimulus space and the distance effect might cause an artifact size effect: Cells from the middle part of the size range include more large-distance cells than cells from the end part of the size range do. Linear slope was fitted both on the error rates and on the reaction times for both the distance and size effects for all participants, then the slopes were tested against zero. Because the simple linear regression analysis gave the very same pattern as the multiple linear regression for all experiments of the present work, the results of this analysis are not presented here.

### Results and Discussion

#### Fitting the Functions of the ANS and the DSS Models to the Reaction Times

**FIGURE 4:**
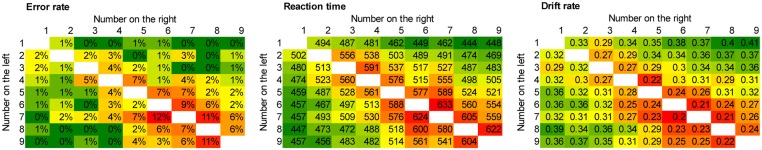
**Error rates (left), response times in ms (middle) and drift rates (right) in the Indo-Arabic digits number comparison for the whole stimulus space.** Green denotes fast and error-free responses, red denotes slow and erroneous responses. Results show distance and size effects.

For the reaction time analysis median reaction time of the correct responses for each number pair and for each participant was calculated. The mean of the participants data for all number pairs (**Figure 4**) were fit linearly with the least square method. Four models were fit to the group mean: The Moyer and Landauer version of the ANS function, the Crossman version of the ANS function, the linear DSS function, and the logarithm DSS function (see Methods for their descriptions).

For the Moyer and Landauer version the data showed a quite appropriate fit, with *R^2^* = 0.884, *AIC* = 613.8, while the Crossman version of the ANS function fit was somewhat worse, although similar, with *R^2^* = 0.769 and *AIC* = 663.5. Regarding the DSS models, the fit for the linear version was *R^2^*= 0.808, *AIC* = 652.4, and the fit for the logarithm version was *R^2^* = 0.893, and *AIC* = 610.3.

Overall, fitting the functions of the four versions of the two models resulted in similar AICs within the same range, therefore no clear preference for any model can be pronounced. It seems that either the appropriate function is not precise enough to have a higher fit (which could be true for either the ANS or the DSS model), and/or with the current noise of the data the subtle differences between the models cannot be investigated. Thus, reaction time analysis with the current functions and the available signal-to-noise ratio could not be decisive in contrasting the ANS and DSS model.

#### Fitting the Functions of the Models to the Error Rates

For the error rate analysis, the mean error rate for each number pair and for each participant was calculated, then the average of the participants was computed (**Figure 4**). To test the ANS model, first, we looked for the Weber ratio that gives the same mean error rate for the stimulus space used here (all possible number pairs for numbers between 1 and 9, ties excluded) as it was measured in our data (2.5%). The found 0.11 Weber ratio was used to generate the predictions of the ANS model for all cells of the stimulus space (see Methods for the function), and the model was linearly fit to the error rate data with the least square method. The goodness of fit was *R^2^* = 0.625, *AIC* = -371. In testing the DSS model, the goodness of fit for the linear version was *R^2^*= 0.505, *AIC* = -341, and the logarithmic DSS model gave a goodness of fit of *R^2^* = 0.667, *AIC* = -377.

Like in the case of the reaction time, the goodness of fit of the ANS and the DSS models are indistinguishable in the error rates data. This again shows that with the signal-to-noise ratio of the present data, the two models are indistinguishable, or the DSS model is not precise enough to show a higher fit.

#### Fitting the Functions of the Models to the Drift Rates

To recover the drift rates for all number pairs in the two notations, the EZ diffusion model was applied, which can be used when the number of trials per cells is relatively small (Wagenmakers et al., 2007). For edge correction we used the half trial solution (see the exact details about edge correction in Wagenmakers et al., 2007). The scaling within-trials variability of drift rate was set to 0.1 in line with the tradition of the diffusion analysis literature. Drift rates for each number pair and participant were calculated in both notations. The mean drift rates of the participants for the full stimulus space are displayed in **Figure 4**.

According to the goodness of fit of the models, the ANS model is worse (*AIC* = -140.1) than the DSS model (*AIC* = -332.4 and *AIC* = -348.1 for the linear and logarithmic DSS model versions, respectively). (Because in a linear fit with zero intercept, the *R*^2^ is much higher than in a linear fit with non-zero intercept (as a consequence of some of the mathematical properties of *R*^2^), and because the ANS model uses 0 intercept, but the DSS model does not, the *R*^2^ values are not reported here.)

Looking at the drift rates of the comparison task (**Figure 4**) might reveal why the ANS model is worse than the DSS model: While the ANS model predicts that the drift rate tends to zero as the stimuli become indistinguishable (e.g., 8 vs. 9), the recovered drift rates are in fact much larger, tending to the 0.2 values. This problem is analogous to a conceptual problem: How is it possible that an imprecise representation solves a precise comparison task? In other words, if the Weber fraction of the ANS is around 0.11, how is it possible that small ratio number pairs, e.g., 8 vs. 9, can still be differentiated with relatively high precision.

Thus, in the diffusion model analysis the DSS model seems to offer a better prediction than the ANS model, however, it is important to note that (a) the EZ diffusion model analysis and more generally any diffusion models have some constrains (Wagenmakers et al., 2007), and consequently, it is possible that in this case the recovered parameters are not entirely reliable, and (b) task difficulty can be defined in different ways (Palmer et al., 2005; Dehaene, 2007), and it might be debated which definition is appropriate. Thus, while the present diffusion model analysis reveals the advantage of the DSS model over the ANS model, the uncertainties of the methods might question how reliable these results are. (The methods and the models are investigated in more details in Krajcsi et al., unpublished).

#### Presence of the Distance and the Size Effects

According to the multiple linear regression analysis, both the distance and the size effects were present both in the error rates and in the reaction times, 95% CI for the slope was [-1.16%, -0.65%], *t*(17) = -7.42, *p* < 0.001 for the distance effect in error rates, and CI of [-23.6 ms, -15.5 ms], *t*(17) = -10.1, *p* < 0.001 in reaction times, CI with [0.3%, 0.59%], *t*(17) = 6.57, *p* < 0.001 for the size effect in error rates, and CI with [4.8 ms, 9.1 ms], *t*(17) = 6.78, *p* < 0.001 in reaction times.

#### Summary

First, we found that reaction time and error rate patterns in Indo-Arabic number comparison (**Figure 4**) could not be decisive in contrasting the ANS and the DSS models. Even if the two models correlate, the correlation is not perfect, and there was a chance that the present test could have decided. Still, with the present models and/or signal-to-noise ratio, the test was not decisive. On the positive side, this means that the DSS model is a viable alternative to the ANS model, because the goodness of fit of the DSS model is in the same range as the goodness of fit of the ANS model. Second, we found that in a diffusion model analysis the drift rate pattern is more in line with the DSS model than with the ANS model, although the uncertainties about the method may question the reliability of these results. Overall, while the performance in the Indo-Arabic comparison task suggests that the DSS model is a viable model, this paradigm could not decide firmly which model is preferred. Thus, in the next experiment a new approach is utilized in which we investigate the role of the frequency in the size effect.

## Experiment 2 – Role Of The Frequency In The Size Effect

In a different approach, we tested whether the distance and the size effects are strongly related as suggested by the ratio-based ANS model, or whether the two effects can dissociate. In the present experiment we investigated whether size effects can dissociate from distance effect if the frequency of the symbols is manipulated. (See another type of test for the dissociation of the two effects in Krajcsi, 2016) To manipulate the frequency of the symbols, it might be more appropriate to use new symbols, instead of the well-known Indo-Arabic symbols, because the frequency of the already known symbols might be well established and learned.

**FIGURE 5:**
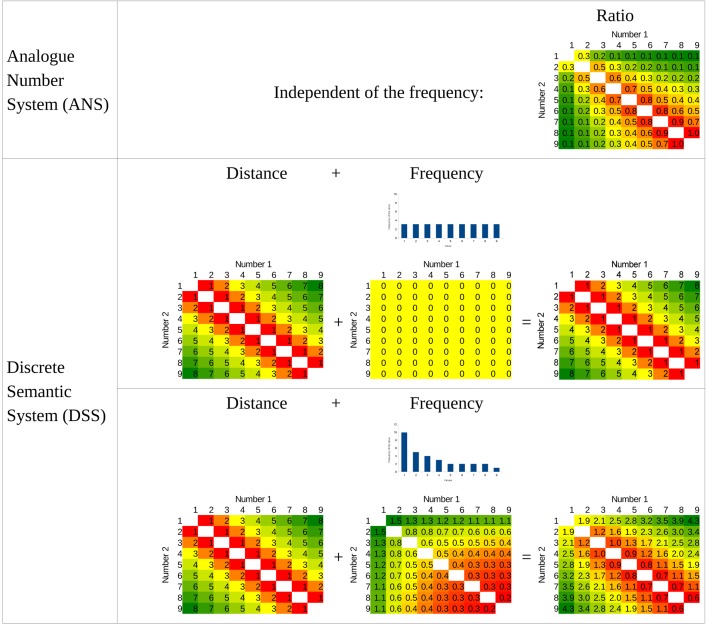
**Prediction of the two models for the symbol frequency manipulation in Experiment 2.** Bar charts show the frequency of the stimuli used in the uniform distribution condition and in the Indo-Arabic-like distribution condition. (In the Indo-Arabic-like distribution the resulting performance is computed as 0.4 × Distance + Frequency.)

Thus, to investigate the role of the frequency in the size effect, participants learned new number symbols in a simple number comparison task, and the frequency of the symbols was manipulated in the experiment. According to the DSS model, the size effect could be changed as a function of the symbol frequencies (**Figure 1**), if the reaction time depends on the frequency of the symbol, and not the frequency of the concept. For example, if the distribution of the frequencies is uniform, then according to the DSS model, the size effect should vanish. In contrast, according to the ANS model, even with uniform frequency distribution the size effect should be visible, because the size effect is rooted in the ratio of the two values, independent of the frequency (**Figure 5**). It is important to stress that although according to the ANS model it might be possible that the frequency of the symbols have an effect on the performance, the effect should be relatively weak: Although in the ANS model the role of the frequency is not discussed, it states that the largest part of the performance variance should be explained by the ratio (Moyer and Landauer, 1967; Dehaene, 2007), which means that any other factors could have only a minor effect on the performance.

### Methods

**FIGURE 6:**
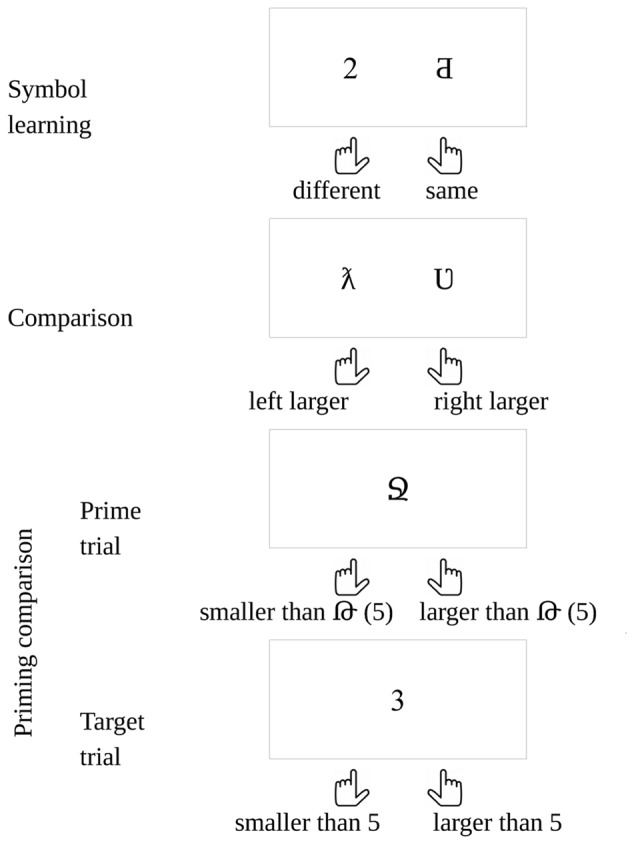
**Tasks in the new symbol experiments**.

Participants learned new symbols (**Figure 6**) for the numbers between 1 and 9 to compare, while the frequency of all symbols was manipulated in two conditions.

It is possible that the new symbols are not connected to the numerical values they represent, and they may be processed only as a non-numerical ordered series. This could cause a problem, because the ANS could not process this non-numerical order^5^. To ensure that the new symbols were connected to the numerical values they represent, at the end of the experiment we used a priming task to measure the priming distance effect (PDE) between the newly learned symbols and familiar Indo-Arabic digits (**Figure 6**). In a PDE the reaction time to the target is faster when the numerical distance between the prime and the target is smaller, reflecting a semantic relation between the prime and the target (Koechlin et al., 1999; Reynvoet and Brysbaert, 1999; Reynvoet et al., 2009).

#### Participants

Eighteen university students participated in the uniform frequency distribution condition. After excluding 2 of them because the error rate did not fall below 5% even after the 5th block, and excluding 2 further participants showing higher than 5% error rates in the main comparison task, the data of 14 participants was included (11 females, mean age 20.6 years, standard deviation 2.1 years). Fifteen university students participated in the Indo-Arabic-like frequency distribution condition. After excluding two participants because their error rates were higher than 5% either in the main comparison or in the priming comparison task, the data of 13 participants was analyzed (13 females, mean age 24.3 years, standard deviation 6.9 years).

#### Stimuli and Procedure

The participants first learned new symbols for the numbers between 1 and 9. Then, in a comparison task they decided which number is larger in a simultaneously presented symbol pair. Finally, in a priming comparison task the participants decided whether one-digit numbers are smaller or larger than 5 (**Figure 6**).

New symbols were introduced to represent values between 1 and 9. The new symbols were chosen from writing systems that were mostly unknown to the participants, and the characters had similar vertical and horizontal size. The symbols were randomly assigned to values for all participants, i.e., the same symbol could mean a different value for different participants, from the following characters: 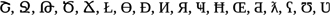.

To ensure that the participants have learned the symbols, in the symbol learning phase, the symbols were practiced until a threshold hit rate was reached. In a trial of the new symbol learning phase a new symbol and an Indo-Arabic digit were shown simultaneously, and the participant decided whether the two symbols denote the same value. The stimuli were visible until response. After the response, auditory feedback was given. In a block all symbols were presented 10 times (90 trials in a block) in a randomized order. In half of the trials the symbols denoted the same values. The symbol learning phase ended if the error rate in a finished block was smaller than 5%, or the participant could not reach that level in five blocks.

In the main comparison task, the same procedure was used as in the first experiment, but here the numbers were denoted with the new symbols. In the uniform frequency distribution condition the number of the presentation of a digit were the same as in the first experiment (all possible number pairs were shown 10 times). In the Indo-Arabic-like frequency distribution condition the frequencies of the specific values followed the frequencies of the numbers in everyday life (Dehaene and Mehler, 1992), specified with the following formula: frequency_value_ = value^-1^ × 10. This formula generated the following frequencies (value:frequency): 1:10, 2:5, 3:4, 4:3, 5:2, 6:2, 7:2, 8:2, 9:1 (**Figure 5**). The 2-permutations of these numbers excluding ties were presented, resulting in 794 trials.

In the priming comparison task in odd (prime) trials a new symbol was visible, and the participant decided whether it was smaller or larger than 5. Two hundred ms after the response in an even (target) trial an Indo-Arabic digit was shown, and the participant decided whether it was smaller or larger than 5. Two thousand ms after the response a new odd (prime) trial was shown. The stimuli were visible until response. The instruction included the value of 5 in both notations: For even trials Indo-Arabic notation (“5”), for odd trials the new notation (e.g., 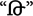) was used. All possible new symbols were presented with all possible Indo-Arabic digits three times, resulting in 192 trials.

### Results and Discussion

**FIGURE 7:**
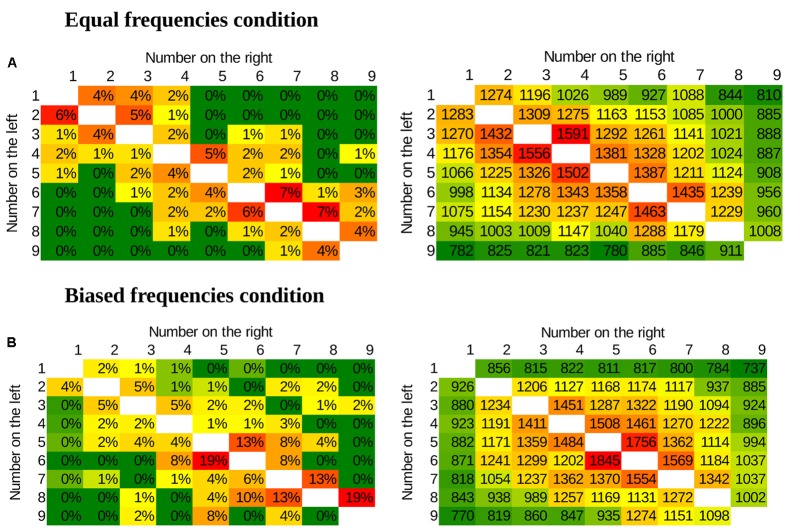
**Error rates (left) and response times in ms (right) in the new symbol number comparison for the whole stimulus space.** Green denotes fast and error-free responses, red denotes slow and erroneous responses. **(A)** Equal frequencies condition, showing distance and end effects. **(B)** Biased frequencies condition, showing distance, size and end effects.

To summarize the main results, in the uniform distribution comparison task the distance effect was present, but the size effect was not (**Figure 7A**). This result is in line with the DSS model, but not with the ANS model. On the other hand, in the Indo-Arabic-like, biased frequency comparison task both the distance and the size effects were visible (**Figure 7B**) in a similar pattern as observable in Indo-Arabic number comparison (**Figure 4**), suggesting that it is the frequency manipulation that is responsible for the size effect.

#### Distance and Size Effects in the Uniform Frequency Distribution

The same analysis methods were applied as in the first experiment with two exceptions. Descriptive data clearly shows an end effect (Leth-Steensen and Marley, 2000). Thus, an end effect regressor was also included in the multiple linear regressions (**Figure 3**) with a value of 1 if any of the presented numbers were 9, 0.5 if any of the numbers were 8 or 1, and 0 otherwise. These values were specified with first calculating the average reaction time for all presented numbers, then the distance effect (distance from 5) of the middle number range (i.e., without end effect) was extrapolated, and finally, the deviation from this extrapolation at the end of the number range was estimated.

In the multiple linear regression the slope of the distance effect deviated from zero, 95% CI was [-1.04%, -0.48%], *t*(13) = -5.84, *p* < 0.001 for error rates, and CI was [-73.6 ms, -26.1 ms], *t*(13) = -4.53, *p* = 0.001 for reaction time. On the other hand, the slope of the size effect did not differ from zero, CI with [-0.15%, 0.06%], *t*(13) = -0.933, *p* = 0.368 for error rates, and CI with [-26.6 ms, 13.9 ms], *t*(13) = -0.679, *p* = 0.509 for reaction time. The end effect was present for the reaction time, CI of [-430.6 ms, -147.6 ms], *t*(13) = -4.41, *p* = 0.001, and more unstably for the error rates, CI with [-0.23%, 2%], *t*(13) = 1.71, *p* = 0.111.

These results also demonstrated an end effect (the most extreme values in the set are easier to respond than other values) (Leth-Steensen and Marley, 2000), however, while the end effect can be in line with the DSS model (Leth-Steensen and Marley, 2000), it is also possible that the effect is irrelevant in the description of the representation processing the numerical values (Balakrishnan and Ashby, 1991; Piazza et al., 2003), consequently, the presence of this effect is not decisive in the present question.

#### Distance and Size Effects in the Indo-Arabic-Like Frequency Distribution

The slope of the distance effect differed from zero in both the error rates, CI with [-1.56%, -0.5%], *t*(12) = -4.25, *p* = 0.001, and in reaction times, [-55.7 ms, -28.9 ms], *t*(12) = -6.87, *p* < 0.001. The non-zero slope of the size effect was also observable, [0.20%, 0.68%], *t*(12) = 3.99, *p* = 0.002 for the error rate, and CI with [28.4 ms, 50.2 ms], *t*(12) = 7.85, *p* < 0.001 for the reaction time. Additionally, the end effect was observable in the reaction times, CI with [-622.5 ms, -294.9 ms], *t*(12) = -6.1, *p* < 0.001, but not in the error rates, CI with [-2.76%, 0.7%], *t*(12) = -1.3, *p* = 0.217.

We tested directly whether the size effects of the two frequency conditions differed. The size effect slopes between the uniform frequency distribution and the Indo-Arabic-like frequency distribution conditions differed significantly in both the error rates, *U* = 13, *p* < 0.001, and in the reaction times, *U* = 15, *p* < 0.001.

#### Priming Distance Effect

**FIGURE 8:**
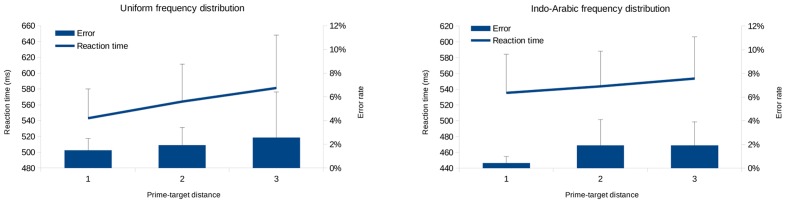
**Prime distance effect (PDE) measured in error rates (bars) and reaction time (lines), in equal frequency condition **(left)** and in biased frequency condition **(right)** in Experiment 2.** Error bars represent 95% confidence interval.

In this analysis the error rates and median reaction times of the correct responses of the target Indo-Arabic numbers were analyzed as a function of the prime (new symbols) – target (Indo-Arabic digit) distance (**Figure 8**). Only the trials in which the response was the same for the prime and distance (i.e., both numbers were smaller than 5, or both numbers were larger than 5) were analyzed (Koechlin et al., 1999; Reynvoet and Brysbaert, 1999; Reynvoet et al., 2009). Linear slope was calculated for the PDE.

In the uniform frequency distribution the data of one participant was not recorded due to technical problems. Because in the symbol learning task participants practiced the new symbol – Indo-Arabic pairs, the zero distance pairs could have this extra practice gain, and not depend purely on the semantic priming effect. Thus, the 0 distance pairs were not included in the analysis. While the descriptive data showed increasing priming effect with smaller distance (**Figure 8**), the effect was not significant: In the uniform frequency condition CI is [-1.62%, 2.69%], *t*(12) = 0.54, *p* = 0.599 for the error rate, and CI is [-1.4 ms, 39.7 ms], *t*(12) = 2.03, *p* = 0.065 for the reaction time, and in the Indo-Arabic frequency condition CI is [-0.13%, 1.63%], *t*(12) = 1.85, *p* = 0.089 for the error rates, and CI is [-8.9 ms, 27.2 ms], *t*(12) = 1.11, *p* = 0.290 for the reaction time. The lack of significance could mean the lack of PDE, or it could reflect the lack of statistical power, or both. Looking at the gradual increase of error rate and reaction time as the function of priming distance (**Figure 8**) and the biased CIs, it seems more probable that the PDE could be statistically significant with larger statistical power. To extend the reasoning that the lack of the significance might be the result of insufficient statistical power, we also analyzed three unpublished similar experiments conducted in our laboratory, where in the same design new symbols were learned with the same stimuli and procedure as in the present works (in the third unpublished experiment the learning and the comparison were repeated for 5 days). In those experiments the PDE was measured with similar sample sizes as in the experiments presented here. We found that in all cases the confidence interval was biased to the direction the PDE predicts, although mostly it was only close to be significant. In the first two unpublished experiments 95% CI is [-6.2 ms, 17.7 ms], *N* = 12, *p* = 0.312, and [29.1 ms, 75.1 ms], *N* = 10, *p* < 0.001. In the third unpublished experiment the PDE was measured for 5 consecutive days which is especially informative about the consistency and fluctuation of the PDE in a relatively small sample: 95% CI [10.25 ms, 34.54 ms], *N* = 13, *p* = 0.002, [-2.09 ms, 28.21 ms], *p* = 0.085, [-1.05 ms, 14.39 ms], *p* = 0.084, [-9.34 ms, 8.13 ms], *p* = 0.882, [-0.49 ms, 13.24 ms], *p* = 0.066, for the five days, respectively. A meta-analysis on the five available experiments (second and third experiments of the present paper and three unpublished experiments; only day 1 was used from the last unpublished experiment; meta-analysis of means in original units with random effect) revealed 95% CI [6.7 ms, 34.3 ms], *p* = 0.004 (Cumming, 2013). The analysis also confirms that the effect size would require much larger sample to have a significant result reliably in a single experiment: The estimated effect size could be as small as *d* = 0.3 (with around 25 ms standard deviation), which would require a magnitude of 100 participants to reach 95% statistical power (Faul et al., 2007). Taken together, based on (a) the expected gradual pattern of the PDE (**Figure 8**), and (b) the consistently biased CIs across experiments, (c) confirmed with the meta-analysis, it is most reasonable to conclude that the PDE is present, even if our usual sample size around 15 does not guarantee the preferred 95% statistical power for a single experiment.

#### Effect of the Frequency

**FIGURE 9:**
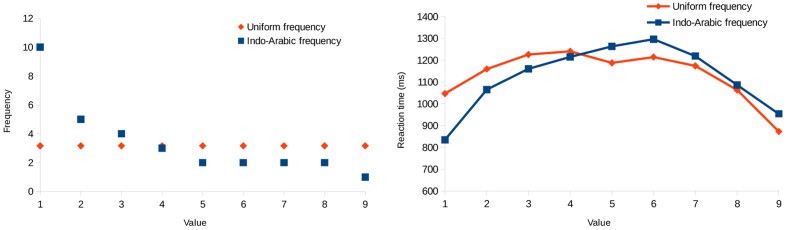
**Frequencies of the specific values (left) and response latencies for those values (right) in Experiment 2**.

To further demonstrate the effect of the frequency (because it cannot be observed readily on **Figure 7**), the mean reaction time was calculated for all cells that include a specific value in both conditions (right of **Figure 9**). The reaction time changes in line with the frequencies of the values: The more frequent a number is in one condition compared to the other condition (left of **Figure 9**), the faster it is to process (right of **Figure 9**). In other words, the differences of the two conditions for the values in the reaction time data are inversely proportional to the differences of the two conditions for the values in the frequency. Note that the reaction time data do not include purely the frequency effect, because (a) middle values are gradually slower to process because of the interaction of the distance effect and the shape of the stimulus space, and (b) end values are faster to process because of the end effect.

#### Summary

In the second experiment the numerical distance and size effects dissociated. More specifically, the numerical size effect was missing when the frequency distribution was uniform, and the size effect was present with the biased frequencies of symbols, suggesting that size effect was guided by the frequencies of the symbols. These results cannot be explained by the ANS model, whereas they can be in line with the DSS model. We highlight again that according to the ANS model although the frequency might slightly modulate the performance, it cannot change a large proportion of the variance in the performance. However, the present result reveal that largest part of the variance of the size effect is directed by the frequency, while the ratio has no observable effect (as revealed by the statistical lack of the size effect), contradicting the ANS model prediction.

Results also show that the new numbers semantically primed the Indo-Arabic digits as revealed by the PDE, demonstrating that the new symbols were connected to the values they represent. Thus, the lack of the size effect in the second experiment cannot be the result of potentially non-numeric new symbols which could not be processed by the ANS.

## Experiment 3 – Role Of The Semantic Congruency Effect In The Size Effect

As another potential confound, it is possible that in the second experiment there was a size effect in the uniform distribution condition, however, a semantic congruency effect (SCE) extinguished it. According to the SCE, large numbers are responded to faster than small numbers when the task is to choose the larger number, resulting in a reversed size-like effect, and small numbers are faster to decide on when the smaller number should be chosen, resulting in a regular size-like effect (Leth-Steensen and Marley, 2000). If the SCE was present in the second experiment, this anti-size effect could have extinguished a potentially existing size effect. To test this possibility, the uniform frequency condition of the second experiment was rerun, but this time the participants had to choose the smaller number. If the SCE was present in the second experiment as a reversed size-like effect, then it should be observed in the present experiment as a regular size-like effect, increasing the size effect. However, the size effect was not present in this control experiment, demonstrating that the SCE did not mask a potentially existing size effect.

### Methods

The methods of the second experiment was applied, however, participants had to choose the smaller number, not the larger, in the comparison task. The priming comparison task was not run.

Eighteen university students participated in the study. Two participants were excluded, because their error rates were higher than 5% after the 5th learning block, and two participants were excluded because they had higher than 5% error rate in the comparison task. The data of 14 participants were analyzed, 10 females, with mean age of 25.4 years, and standard deviation of 6.9.

### Results

#### Distance and Size Effects

In the multiple linear regression analysis the distance effect was present in both the error rate and the reaction time, CI [-1.54%, -0.48%], *t*(13) = -4.13, *p* = 0.001, and CI [-77.0 ms, -39.6 ms], *t*(13) = -6.73, *p* < 0.001, respectively. More critically, the size effect was not observable neither in the error rate nor in the reaction time, CI [-0.18%, 0.15%], *t*(13) = -0.184, *p* = 0.857, and CI [-25.0 ms, 26.2 ms], *t*(13) = 0.0482, *p* = 0.962, respectively. Comparing the slopes of the uniform frequency condition of the second and the present experiments, the slopes of the size effects did not differ significantly, neither in error rate nor in reaction time, t(26) = 0.33, *p* = 0.744, and *U* = 91.5, *p* = 0.783, respectively. Thus, choosing the smaller number did not change the size effect, consequently, the SCE did not influence essentially the size effect in the second experiment.

## General Discussion

We introduced the DSS model as a comprehensive alternative account to the ANS model to explain symbolic number processing. First, we have shown that the DSS model can explain many symbolic numerical effects, and we demonstrated that the DSS model could give a similar quantitative prediction for symbolic number comparison performance as the ANS model. Second, we tried to contrast the two models in Indo-Arabic comparison task. However, because of the relatively high noise and the uncertainties of the diffusion analysis method, it was not possible to find a straightforward preference for any models. On the other hand this result could show that the DSS model prediction empirically fits the Indo-Arabic number comparison as good as the ANS model prediction. Finally, the second and third experiments revealed that in new symbol comparison tasks the numerical size effect is the consequence of the frequency manipulations of the symbols, as proposed by the DSS model, and not the consequence of the ratios of the values, as predicted by the ANS model. These data also show that the numerical distance and size effects are not straightforward signs of the ANS, because an alternative mechanism could produce them as well.

While the second and the third experiments utilized new symbols, it is possible to extend our conclusion about other symbolic number comparisons, for example, the Indo-Arabic number comparison. Because all known numerical effects that were observable in the new symbol comparison show the very same pattern as in Indo-Arabic comparison (i.e., distance effect, size effect and PDE), it is parsimonious to suppose that the same mechanisms work behind new symbol comparison and Indo-Arabic comparison, and our findings can also be generalized to the Indo-Arabic and other symbolic number processing, unless additional data show the opposite.

We argue that the ANS model is not in line with our results. While one can try to modify the ANS model to align with the present result, ratio-based performance is a defining feature of the ANS, and changing that feature leads not only to a modified ANS model, but to a completely new model. Additionally, adding frequency effect to the ANS model cannot modify it to explain the frequency-based size effect, because the ANS critically suggests that the performance should mainly be driven by the ratio, which ratio effect in fact was statistically invisible in the second and third experiments.

We argue that the ANS model is not in line with the present results, and the DSS can be an appropriate alternative. However, one might question how strongly our results support a DSS model. Obviously, one can only tell if a model is in line with the empirical results, and whether the model is coherent. We argue that the DSS is in line with the present and previous results (e.g., it can explain the independent distance and size effects, why symbolic and non-symbolic comparisons are relatively independent, or how arbitrarily precise comparison can be made), and it is a coherent model. Additionally, based on current cognitive models, it is reasonable to suppose that abstract symbolic operations are processed by a system that is otherwise known to be used for other symbolic operations, such as the mental lexicon or a conceptual network. On the other hand, no one can exclude that an alternative, third model could account for these results, and not the ANS or the DSS models. Further research can tell whether the DSS framework is an appropriate explanation for the symbolic number processing or another alternative should be found. Furthermore, it is possible that it is not a single representation that is responsible for the discussed effects, but cooperation of several representations is required, and although the ANS cannot explain the distance effect in comparison task, still there could be other symbolic numerical phenomena that could be rooted in the ANS. Additional works can find out whether such a partial role can be attributed to the ANS in symbolic number processing.

The DSS model in its current form relies on models about mental lexicon or conceptual networks. These starting points could offer many properties of the models, while at the same time, many other details are seemingly missing, e.g., the exact quantitative description of the comparison performance. While these shortcomings might make the impression that the DSS model is less detailed than the ANS model, these differences are the consequence of changing the base of the explanations. While the ANS model is a low-level perceptual model in its nature, the DSS model is more like a linguistic or conceptual network model. Models describing higher level functions are usually less quantitative than models describing lower level functions, partly because of methodological reasons, and from this viewpoint it seems reasonable that a DSS model is less quantitative than an ANS model. However, from a different—and more relevant—viewpoint, the DSS model is as efficient as the ANS model, because seemingly all relevant symbolic numerical effects and phenomena can also be explained in the DSS model, and a few examples can already be found where the DSS model can give a better explanation than the ANS model.

The ANS model is a widely accepted and deeply grounded explanation for number processing. However, despite the huge amount of papers discussing and supporting the ANS view, they are relying on surprisingly few effects and findings that demonstrate an ANS activation. In fact, the few phenomena can also be explained in the alternative DSS model as well. Additionally and more importantly, an increasing number of findings are not in line with the ANS model. For example, symbolic and non-symbolic performance seems to be independent on many behavioral (Holloway and Ansari, 2009; Sasanguie et al., 2014; Schneider et al., 2016) and neural level (Damarla and Just, 2013; Bulthé et al., 2014, 2015; Lyons et al., 2015). In a correlational study it has been shown that distance and size effects dissociate in Indo-Arabic comparison task (Krajcsi, 2016). Some results show that the numerical representation is not analog: Functional activation in the brain while processing symbolic numbers seems to be discrete (Lyons et al., 2015), and symbolic numbers can also interfere with the discrete yes-no responses (Landy et al., 2008). The present finding showing the frequency dependence of the size effect also extends the list of results contradicting the ANS model. Future research can tell whether the ANS can be reformulated to account for these findings, or an alternative model, such as the DSS, can characterize symbolic number processing better.

## Author Contributions

All authors listed, have made substantial, direct and intellectual contribution to the work, and approved it for publication.

## Conflict of Interest Statement

The authors declare that the research was conducted in the absence of any commercial or financial relationships that could be construed as a potential conflict of interest.

## Footnotes

1 Comparison distance effect (e.g., which of two numbers is larger) and priming distance effect (whether previous stimulus influences the actual stimulus processing based on the distance of the two stimuli) are known to be two different mechanisms (Verguts et al., 2005; Reynvoet et al., 2009). While we want to find a DSS explanation for the comparison distance effect, the cited semantic distance effect is more similar to a priming distance effect. Importantly, we are not stating that these two effects are the same, but we suggest that a distance-based effect is possible in a DSS, independent of the exact mechanism behind that effect.

2 A similar proposal is that the numerical distance effect might emerge from the order property of numbers, and a distance effect can be observed not only in numbers, but also in non-numerical orders, e.g., days or letters (Potts, 1972; Verguts and Van Opstal, 2014). However, (a) it might be possible that in those examples the non-numerical orders are transformed to the numerical representation, which is not possible for the categorical words in the cited picture naming task (Vigliocco et al., 2002), and (b) the DSS model has less strict constrains, i.e., no order structure is presupposed, but a more general series of associations is sufficient to explain the distance effect.

3 Frequency is essential in other numerical tasks to produce size effect (Zbrodoff and Logan, 2005), and the role of frequency in size effect was also proposed in other alternative models of number comparison (Verguts and Van Opstal, 2014).

4 After creating additional versions of the DSS quantitative prediction with considering the constrains described here, we found qualitatively similar patterns. See another example in the Methods section of Experiment 1.

5 Note, however, that several works suggest that order processing and quantity processing rely on the same mechanisms (Leth-Steensen and Marley, 2000; Marshuetz et al., 2006; Verguts and Van Opstal, 2014), thus, ANS should be activated even when the new symbols are non-numerical orders.
